# Insights into Molecular Mechanisms of Anticancer Activity of *Juniperus communis* Essential Oil in HeLa and HCT 116 Cells

**DOI:** 10.3390/plants13172351

**Published:** 2024-08-23

**Authors:** Tijana Marković, Suzana Popović, Sanja Matić, Marina Mitrović, Marijana Anđić, Aleksandar Kočović, Milena Vukić, Vladimir Petrović, Jovica Branković, Nenad Vuković, Danijela Todorović, Miroslava Kačániová, Dejan Baskić

**Affiliations:** 1 Department of Pharmacy, Faculty of Medical Sciences, University of Kragujevac, Svetozara Markovića, 69, 34000 Kragujevac, Serbia; tianafeels@gmail.com (T.M.); andjicmarijana10@gmail.com (M.A.); salekkg91@gmail.com (A.K.); 2 Centre for Molecular Medicine and Stem Cell Research, Faculty of Medical Sciences, University of Kragujevac, Svetozara Markovića, 69, 34000 Kragujevac, Serbia; popovic007@yahoo.com (S.P.); dejan.baskic@gmail.com (D.B.); 3 Department of Medical Biochemistry, Faculty of Medical Sciences, University of Kragujevac, Svetozara Markovića, 69, 34000 Kragujevac, Serbia; mitrovicmarina34@gmail.com; 4 Department of Chemistry, Faculty of Science, University of Kragujevac, Radoja Domanovića, 12, 34000 Kragujevac, Serbia; milena.vukic@pmf.kg.ac.rs (M.V.); vladimir.petrovic@pmf.kg.ac.rs (V.P.); jovica.brankovic@pmf.kg.ac.rs (J.B.); nenad.vukovic@pmf.kg.ac.rs (N.V.); 5 Department of Genetics, Faculty of Medical Sciences, University of Kragujevac, Svetozara Markovića, 69, 34000 Kragujevac, Serbia; dtodorovic197@gmail.com; 6 Institute of Horticulture, Faculty of Horticulture and Landscape Engineering, Slovak University of Agriculture, Tr. A. Hlinku 2, 94976 Nitra, Slovakia; miroslava.kacaniova@gmail.com; 7 School of Medical & Health Sciences, University of Economics and Human Sciences in Warsaw, Okopowa 59, 01 043 Warsaw, Poland; 8 Institute of Public Health Kragujevac, 34000 Kragujevac, Serbia

**Keywords:** *Juniperus communis*, apoptosis, cancer

## Abstract

As cancer remains a significant global health challenge, there is an increasing need for novel therapeutic approaches. We investigated the antitumor potential of *Juniperus communis* berry essential oil on cervical cancer HeLa and colorectal HCT 116 cells. Cytotoxicity was evaluated through the MTT assay, revealing concentration-dependent reductions in cell viability. A clonogenic assay demonstrated long-term cytotoxic effects. Apoptosis markers were assessed via flow cytometric analysis and showed an induction of the intrinsic pathway in both cell lines, demonstrated by the elevated levels of cleaved caspase-3, Bax/Bcl-2 ratio, JC-10 monomer formation, and cytochrome C migration to the cytosol. The treatment inhibited cell-survival pathways in HCT 116 cells and arrested HeLa cells in the S phase. An extensive molecular docking screening provided insight into the binding affinity and interaction patterns of the essential oil components with NADH ubiquinone oxidoreductase and superoxide dismutase enzymes, further confirming the induction of the intrinsic pathway of apoptosis. The obtained in silico and in vitro results indicated the anticancer potential of *J. communis* berry essential oil as it interferes with cancer cell molecular mechanisms. Our findings highlight *J. communis* berry essential oil as a promising natural agent with anticancer potential.

## 1. Introduction

Cancer is one of the main public health problems of the 21st century. According to the WHO, it is a leading cause of death worldwide, resulting in almost ten million deaths in 2020 [1]. The problem lies in the pathogenesis of cancer, which is dynamic and perplexing. To this day, research for various anticancer therapies is still ongoing. Scientists face many challenges including genetic, epigenetic, and environmental factors affecting cancer progression. Moreover, emerging drug resistance and the serious adverse effects of conventional antitumor therapy enforce the search for new therapeutic approaches, primarily the research of natural substances. Historically, the use of more than three thousand plant species was documented with evidence of antitumor potential. At the moment, there are four classes of plant-derived compounds used in antitumor therapy, including vinca alkaloids, epipodophyllotoxin derivatives, camptothecin analogs, and taxane diterpenoids [2]. In recent years, a focus has been set on discovering novel biologically active compounds with improved efficacy, selectivity, as well adjuvant potential for conventional antitumor therapy. Essential oils (EOs) have emerged as promising candidates, demonstrating a broad spectrum of biological effects, such as antimicrobial, antioxidative, immunomodulatory, and antitumor activity [3,4,5]. The effect of EOs depends on the vast number of active metabolites they contain. The number of different low-molecular-weight chemical compounds in their composition can vary depending on numerous extrinsic and intrinsic factors, with the most important being the extraction process, the plant’s environment, and harvest time [6]. This variability in composition makes it challenging to identify the exact pathways in the body that are affected by EOs. Nevertheless, their incorporation into many consumer products and emerging scientific data highlight their versatility and widespread acceptance.

*Juniperus communis*, commonly known as “kleka” in the Balkan region, is a versatile and widely distributed coniferous plant belonging to the family of Cupressaceae. It is widely distributed through the northern hemisphere, with North America, Europe, and Asia having the most widespread *Juniperus* species. It is known that *J. communis* berries are a rich source of many biologically active metabolites, mostly from the monoterpene family, like α-pinene, sabinene, limonene, myrcene, and β-pinene [7,8].

Therefore, the aim of this study was to examine the molecular mechanism of the anticancer potential of *J. communis* berry essential oil. Our findings could provide insight into the significant anticancer potential of the *J. communis* essential oil in vitro and further help improve knowledge and spike interest in the possible application of other essential oils rich in biologically active compounds in the treatment of cancer.

## 2. Results

### 2.1. J. communis Essential-Oil-Induced Selective Short-Term Cytotoxic Effects in HeLa and HCT 116 Cells

The short-term cytotoxic activity of *J. communis* EO was evaluated with a 3-(4,5-dimethylthiazol-2-yl)-2,5 diphenyltetrazolium bromide dye (MTT) colorimetric assay on three cell lines—nontransformed human lung fibroblasts (MRC-5), human cervical cancer cells (HeLa), and human colon cancer cells (HCT 116). The results are represented graphically in Supplementary Figure S1. The cells were treated at various concentrations (0.1, 0.3, 1, 3, 10, 30, and 100 µg/mL), and cytotoxicity was measured after 24, 48, and 72 h of treatment. *J. communis* EO showed a statistically significant cytotoxic effect in a concentration-dependent manner on every cell line (at 30 µg/mL and 100 µg/mL, *p* < 0.05); however, a statistically significant cytotoxic effect was observed at 10 µg/mL after 48 h only on the HeLa cell line (*p* < 0.05). Moreover, a statistically significant difference in cytotoxicity was observed between the transformed HeLa cell line and the non-transformed MRC-5 cell line at a dose of 30 µg/mL after 48 h of treatment (*p* < 0.001). Doxorubicin, an approved cytotoxic drug, was employed to treat cells in order to serve as a positive control for the MTT experiment (Supplementary Figure S2).

The half-maximal inhibitory concentration (IC_50_) and selectivity index (SI) were calculated to determine the overall inhibitory potential of the EO, with no reference to the initial cell number (Table 1). The obtained data showed that the treatment induced the strongest inhibition against the HeLa cell line, as marked by a low IC_50_ value after 24, 48, and 72 h followed by the HCT 116 cell line. A statistically significant difference between the MRC-5 and HeLa IC_50_ values was observed after every time point, and additionally, there was a statistically significant difference between the HeLa and HCT 116 IC_50_ values at the 48 h point. The *J. communis* EO showed selectivity for malignant cells compared to the non-transformed MRC-5 cells, with the HeLa cell line being the most susceptible to treatment with the highest SI, followed by the HCT 116 cell line. The cytostatic (i.e., 50% cell growth inhibition—GI_50_ and total growth inhibition—TGI) and cytocidal effect (i.e., median lethal dose—LD_50_) were calculated considering the count of cells at time zero. A statistically significant difference in cell proliferation—GI_50_ values—was observed between the MRC-5 and HeLa cell line after 48 h (*p* < 0.05), with the *J. communis* EO exhibiting a significant cytostatic effect on the HeLa cell line compared to the non-transformed cell line. The difference was also significant (*p* < 0.05) between the GI_50_ of HeLa and HCT 116 after 48 and 72 h. A statistically significant difference in TGI was observed at every time point between the MRC-5 and HeLa cell line (*p* < 0.05), while the difference between the two transformed cell lines was observed only after 72 h. The LD_50_ values after 24 and 48 h on the MRC-5 and HeLa cell lines were higher than 100 µg/mL. The HCT 116 cell line had lower LD_50_ values. After 72 h, a statistically significant difference in the LD_50_ was observed between MRC-5 and HeLa, as well as HeLa and HCT 116, implying that the HeLa cell line responded to the lowest median lethal dose between the tested cell lines.

### 2.2. J. communis Essential-Oil-Induced Long-Term Cytotoxic Effects in HeLa and HCT 116 Cells

**Figure 1:**
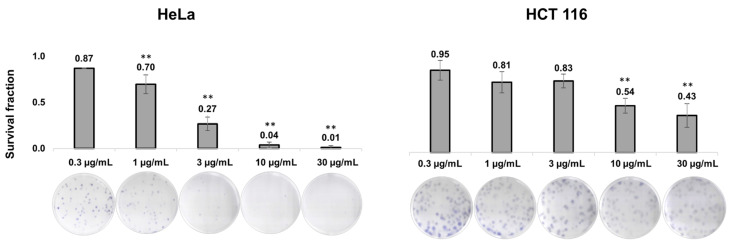
Clonogenic survival of HeLa and HCT-116 cells treated with various concentrations of *J. communis* essential oil. Bar graphs show the survival fraction of HeLa and HCT 116 cells. Representative images of the clonogenic assay below the graphs correspond to treatment concentrations. Results are from three separate experiments presented as mean ± SD. Statistical significance compared to the control (non-treated) group—** *p* < 0.001.

After the MTT assay, the next step was to explore the long-term effects of the *J. communis* EO. The results are presented in Figure 1 as values of the survival fraction (SF). The obtained results from the 12-day clonogenic assay suggested that the EO significantly decreased the SF of the investigated cancer cells in a dose-dependent manner. Specifically, on the HeLa cell line, treatment with concentrations 1, 3, 10, and 30 µg/mL showed a statistically significant decrease in the SF (*p* < 0.001) in comparison to the control (the non-treated group), but also between the treatments. On the HCT 116 cell line, the SF significantly decreased at higher treatment concentrations of 10 and 30 µg/mL, in comparison to the non-treated group. Therefore, a long-term cytotoxic effect was confirmed on both cancer cell lines.

### 2.3. J. communis Essential-Oil-Induced Apoptosis in HeLa and HCT-116 Cells by Upregulating the Expression of Active Caspase-3 and Bax/Bcl-2 Protein Ratio

**Figure 2:**
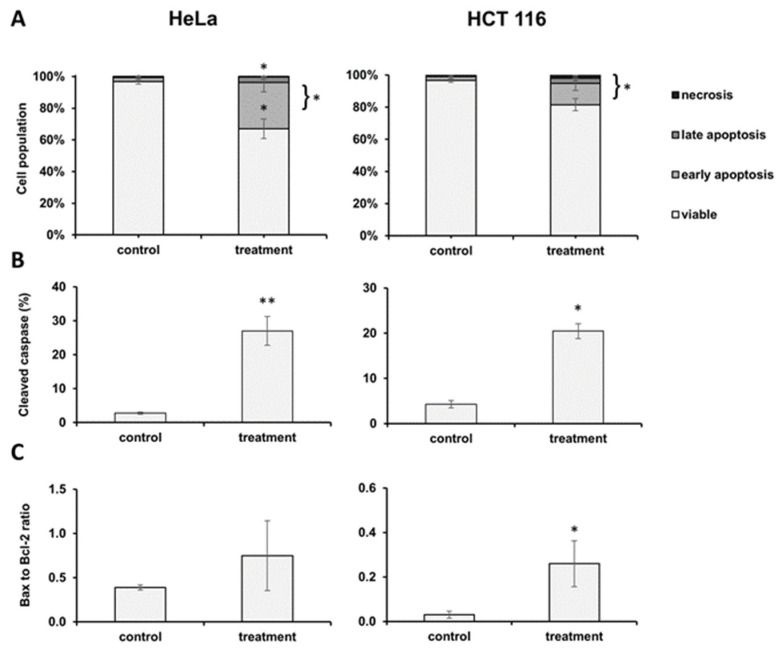
Parameters of apoptosis after treatment with *J. communis* essential oil IC_50_ values following a 48 h incubation. (**A**) Apoptotic cell death in HeLa and HCT 116 cells. Percentage distribution of early apoptotic, late apoptotic, and necrotic cells on the bar charts. (**B**) Ratio of Bax to Bcl-2 protein expression in HeLa and HCT 116 cell line. (**C**) Percentage of active caspase-3 in HeLa and HCT 116 cell lines. Data are expressed as mean ± SD from three independent experiments, each performed in triplicates. Statistical significance compared to control (non-treated) group—* *p* < 0.05; ** *p* < 0.001.

After 48 h incubation with the IC_50_ values of the EO, we performed the flow cytometric analysis of apoptosis using Annexin V-FITC/7-AAD. The results are presented in Figure 2A as percentages of early apoptotic (EA), late apoptotic (LA), and necrotic cells (N). In every group, control and treatment, a fraction of necrotic cells was negligible.

On the treated HeLa cell line, the percentage of EA cells was higher than in the control cells (*p* < 0.05), as well as the portion of LA cells (*p* < 0.05). Comparing the total apoptosis percentage (EA + LA), a statistically significant change was observed between the control and treated HeLa cells. On the HCT 116 cell line, the percentage of EA and LA cells in the control versus treated cells was not significantly different, but the total percentage of apoptosis was significantly higher than the control (*p* < 0.05). In both the HeLa and HCT 116 cell lines, there was a statistically significant increase in the percentage of cleaved caspase-3 by 9.81-fold, *p* < 0.001, and 4.77-fold, *p* < 0.05, respectively (Figure 2B). Additionally, a flow cytometric analysis was performed to further confirm the pathway of apoptotic cell death. We calculated the ratio between pro-apoptotic Bax and antiapoptotic Bcl-2 protein. As shown in Figure 2C, on both cell lines, the Bax/Bcl-2 ratio was higher in the EO-treated cells than in the control, but a statistically significant difference was observed in the HCT 116 cells (*p* < 0.05).

### 2.4. J. communis Essential Oil Treatment Altered the Mitochondrial Membrane Potential in HeLa and HCT 116 Cell Line

**Figure 3:**
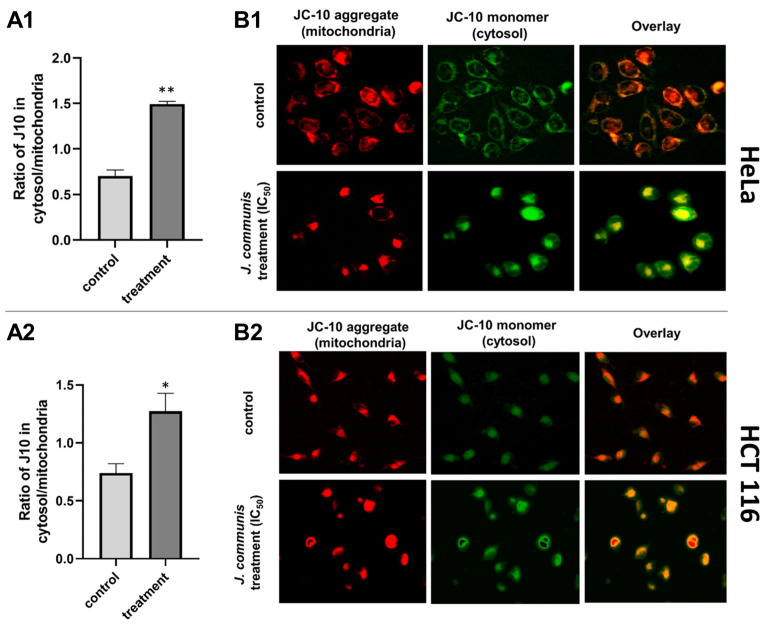
Analysis of mitochondrial membrane potential alterations using JC-10 in HeLa and HCT 116 cells treated with *J. communis* EO. (**A1**,**A2**)—graphical representation of the JC-10 ratio in cytosol versus mitochondria; (**B1**,**B2**)—representative images from fluorescence microscopy. Statistical significance compared to the control (non-treated) group—* *p* < 0.05; ** *p* < 0.001.

**Figure 4:**
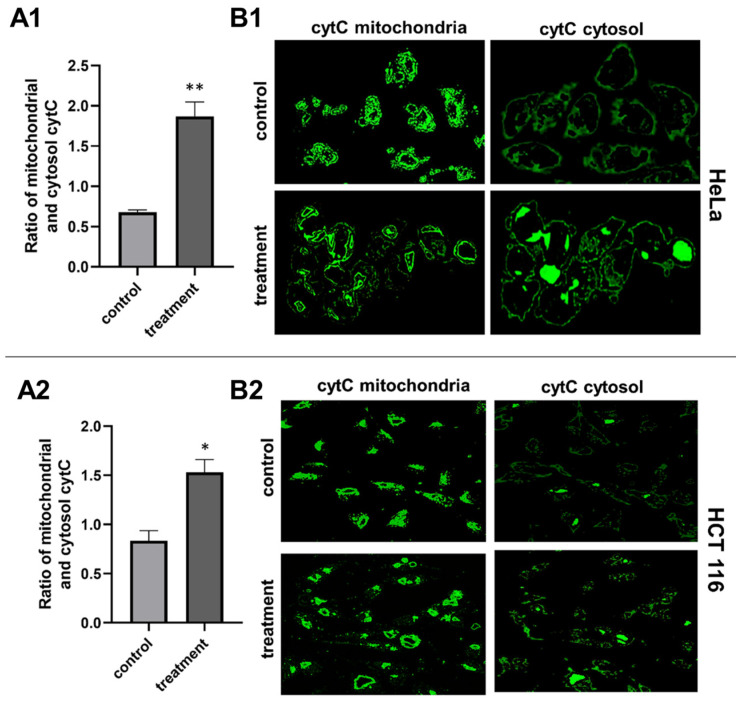
Quantification of cytosol and mitochondrial cytochrome C. (**A1**,**A2**)—graphical representation of the cytochrome C ratio in cytosol versus mitochondria. (**B1**,**B2**)—representative images from fluorescence microscopy. Statistical significance compared to the control (non-treated) group—* *p* < 0.05; ** *p* < 0.001.

To assess the apoptosis pathway that the *J. communis* EO initiates, we investigated the alterations in the mitochondrial membrane potential (ΔΨm)—the indicator of mitochondrial dysfunction. This experiment was conducted with immunofluorescence techniques using the fluorescent dye JC-10. As shown in Figure 3, on the HeLa cell line, the ratio of cytosol to mitochondrial JC-10 (monomer/aggregate) was significantly higher in the treated cells (*p* < 0.001), as well as on the HCT 116 cell line (*p* < 0.05). The change from red to green fluorescence indicated that the treatment causes the reduction in the ΔΨm. We investigated the localization of cytochrome C to further confirm the mitochondrial cell death pathway (Figure 4). In the HeLa cell line, the ratio of cytosol to mitochondrial cytochrome C is significantly higher in the treated cells (*p* < 0.001), as well as in the HCT 116 cell line (*p* < 0.05).

### 2.5. J. communis Essential Oil Treatment Inhibited MAPK/ERK and PI3K/Akt Cell Signaling Pathways of Cell Survival in HCT 116 Cell Line

**Figure 5:**
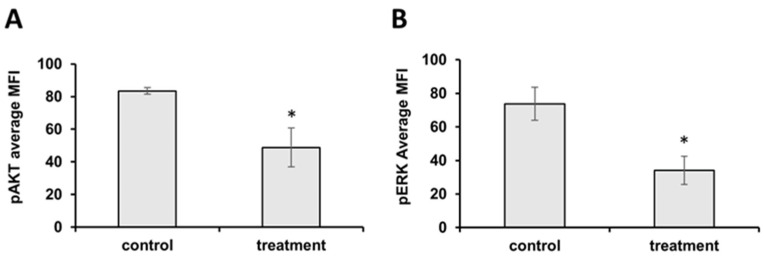
(**A**) Average MFI values for phosphorylated AKT on HCT 116 cells. (**B**) Average MFI values for phosphorylated ERK on HCT 116 cells. Statistical significance compared to the control (non-treated) group—* *p* < 0.05.

To further investigate the apoptotic pathway on the HCT 116 cell line, we conducted a flow cytometric analysis of the MAPK/ERK and PI3K/AKT cell-survival pathways. The results showed that the *J. communis* EO treatment significantly downregulated the expression of both pAKT and pERK, and therefore inhibited the cell-survival pathways, directly resulting in the induction of apoptosis (Figure 5A,B).

### 2.6. J. communis Essential Oil Treatment Affected the Cell-Cycle Distribution in HeLa and HCT 116 Cells

**Figure 6:**
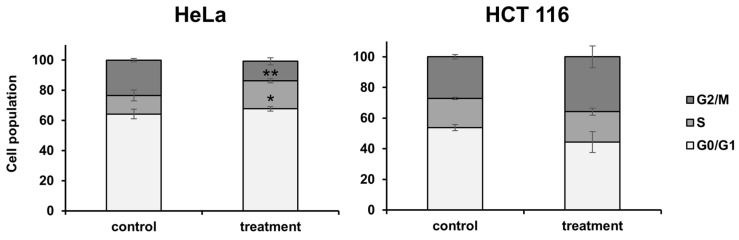
Analysis of cell-cycle distribution in HeLa and HCT 116 cell lines post-IC_50_ treatment and 48 h incubation. Statistical significance compared to the control (non-treated) group—* *p* < 0.05; ** *p* < 0.001.

The cell-cycle distribution was analyzed by measuring the FL4 intensity (PI) using a flow cytometer after 48 h of IC_50_ treatment. As shown in Figure 6, on the HeLa cell line, the results showed that the percentage of cells in the S phase increased by 1.49-fold (*p* < 0.05) and the G2/M phase decreased by 1.8-fold (*p* < 0001). The treatment on the HCT 116 cell line did not significantly affect the cell-cycle distribution (*p* > 0.05), although a slight change in the G2/M was observed.

### 2.7. J. communis Essential Oil Active Metabolites Bound to the NADH Ubiquinone Oxidoreductase Subunits 1, 2, 6 and Superoxide Dismutase

**Figure 7:**
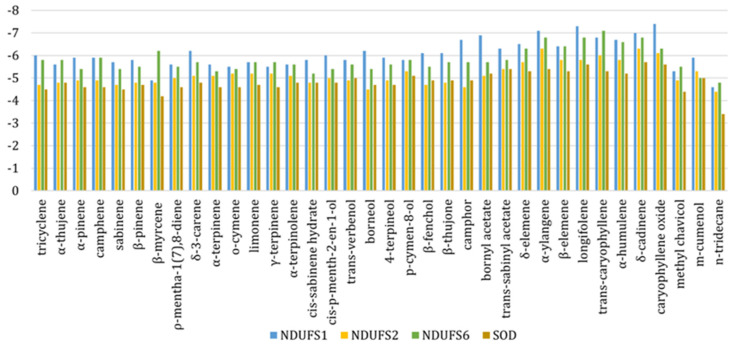
Calculated binding-affinity values (kcal/mol) of *J. communis* EO components for NDUFS1, NDUFS2, NDUFS6, and SOD.

The binding modes of the *J. communis* EO components were investigated in silico against NADH ubiquinone oxidoreductase (complex I-CI) subunits S1, S2, and S6 (NDUFS1, NDUFS2, and NDUFS6) and superoxide dismutase (SOD). Based on the obtained results, the binding-affinity values were calculated in the range from −3.4 to −7.4 kcal/mol, as shown in Figure 7.

**Figure 8:**
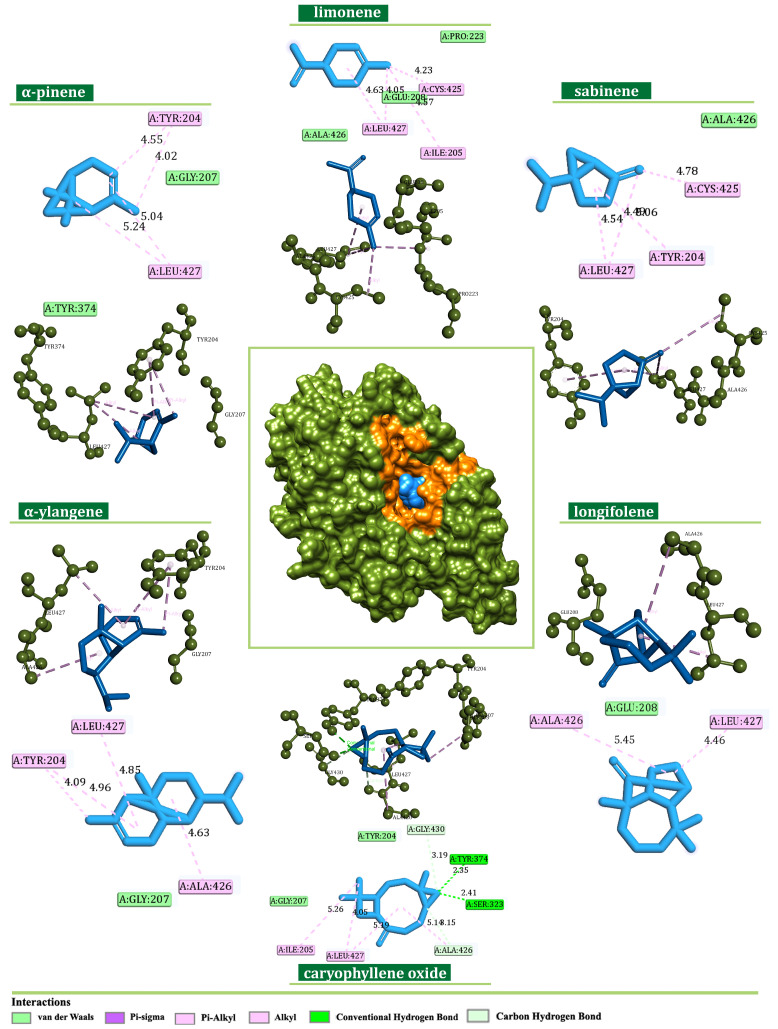
Binding modes of the best-screened *J. communis* EO components (blue) with NADH ubiquinone oxidoreductase subunit S1 (NDUFS1) presented with 2D interaction plots.

The investigation of the binding potential toward NDUFS1 revealed favorable interaction patterns of α-pinene (−5.9 kcal/mol), limonene (−5.7 kcal/mol), and sabinene (−5.7 kcal/mol). On the other hand, α-ylangene (−7.1 kcal/mol), longifolene (−7.3 kcal/mol), and caryophyllene oxide (−7.4 kcal/mol) were identified as the most prominent binders of NDUFS1 among minor constituents. All ligands occupied the CASTp-predicted active-site region forming hydrophobic alkyl, π-alkyl, and π-sigma interactions with Tyr204, Leu427, Cys425, Ile205, and Ala426 (Figure 8). Moreover, the formation of two hydrogen bonds with Tyr374 (dHB = 2.35 Å) and Ser323 (dHB = 2.41 Å) was identified in the case of caryophyllene oxide.

**Figure 9:**
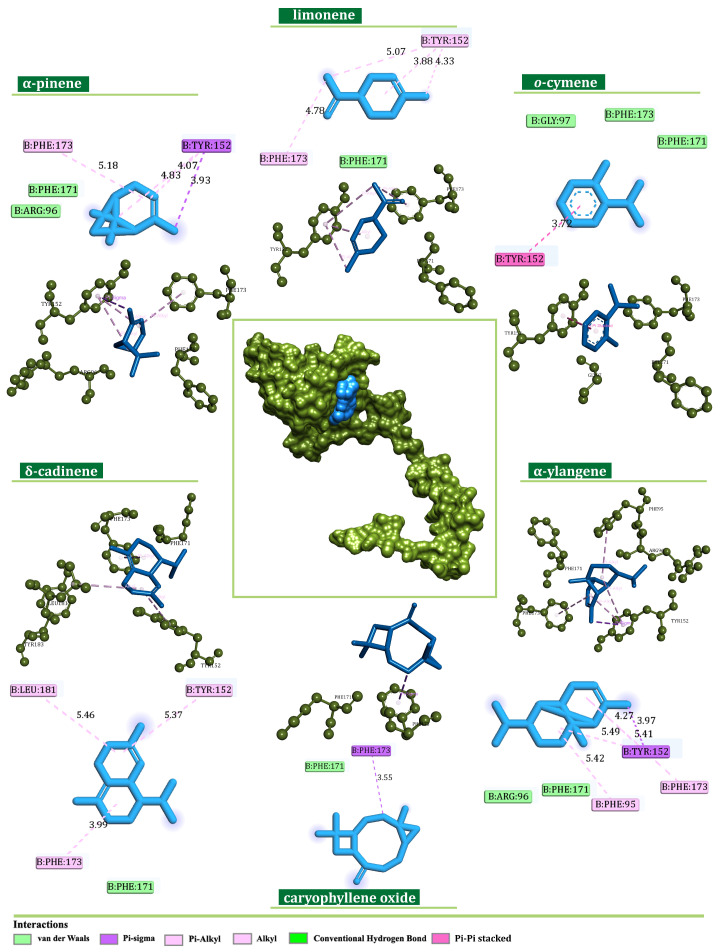
Binding modes of the best-screened *J. communis* EO components (blue) with NADH ubiquinone oxidoreductase subunit S2 (NDUFS2) presented with 2D interaction plots.

Similarly, the docking simulations performed on NDUFS2 exposed δ-cadinene (−6.3 kcal/mol), α-ylangene (−6.3 kcal/mol), and caryophyllene oxide (−6.1 kcal/mol) as components with the highest binding affinity. On the other hand, α-pinene (−4.9 kcal/mol), limonene (−5.2 kcal/mol), and o-cymene (−5.2 kcal/mol) were identified as the most prominent among major components. The corresponding ligands interacted within the predicted active site with Phe95, Tyr152, Tyr173, and Leu181 via alkyl, π-alkyl, π-sigma, and π-π hydrophobic interactions, respectively, as shown in Figure 9.

**Figure 10:**
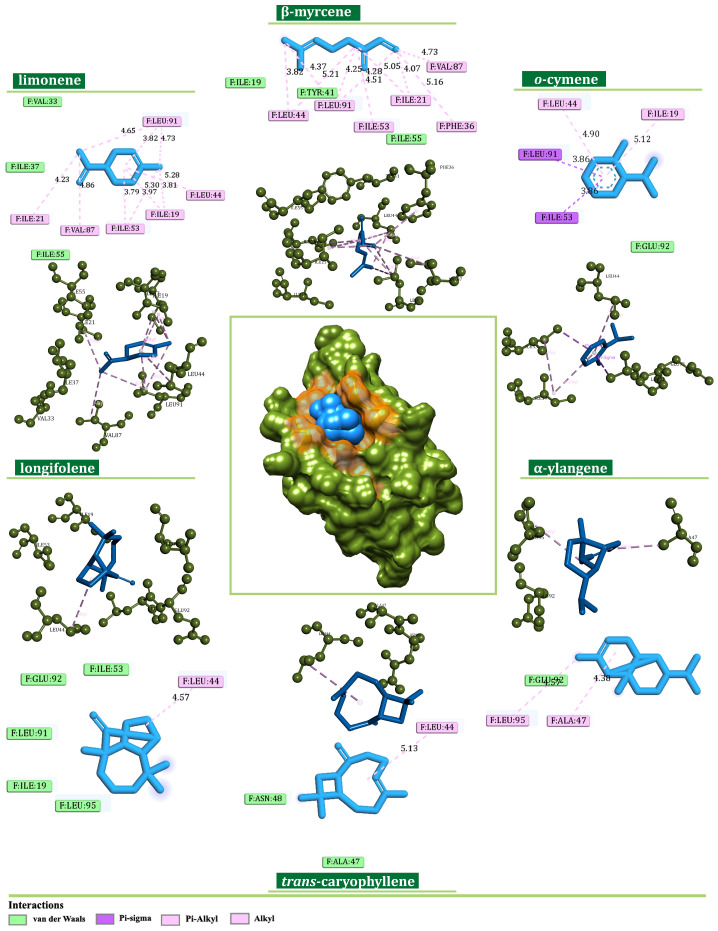
Binding modes of the best-screened *J. communis* EO components (blue) with NADH ubiquinone oxidoreductase subunit S6 (NDUFS6) presented with 2D interaction plots.

The investigated EO components expressed significant potential to bind NDUFS6 also. Here, β-myrcene (−6.2 kcal/mol), limonene (−5.7 kcal/mol), *o*-cymene (−5.4 kcal/mol), longifolene (−6.8 kcal/mol), α-ylangene (−6.8 kcal/mol), and *trans*-caryophyllene (−7.1 kcal/mol) established multiple alkyl, π-alkyl, and π-sigma interactions with amino acid residues of Ile21, Ile19, Ile53, Val87, Leu44, Leu91, Phe36, Ala47, and Leu95 (Figure 10).

**Figure 11:**
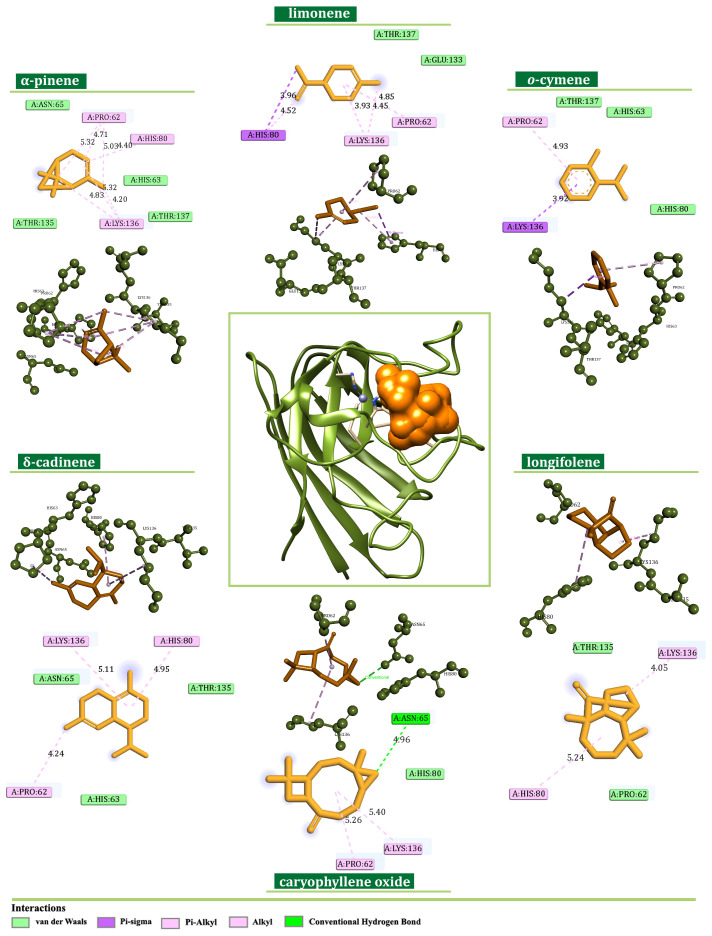
Binding modes of the best-screened *J. communis* EO components (orange) with superoxide dismutase SOD presented with 2D interaction plots.

In the case of SOD, δ-cadinene, caryophyllene oxide, and longifolene were identified as the most effective binders among minor EO components (−5.7, −5.6, and −5.6 kcal/mol, respectively). On the other hand, α-pinene, o-cymene, and limonene expressed the highest binding affinity toward SOD among major constituents, with energy values of −4.5, −4.6, and −4.6 kcal/mol. A more profound comprehension of the binding modes was achieved by an interaction pattern analysis (Figure 11). Here, all ligands were positioned within the predicted active-site region establishing predominantly alkyl, π-alkyl, and π-sigma interactions with the side chains of Pro62, His80, and Lys136.

### 2.8. J. communis Essential Oil Treatment Decreased the O_2_^•−^ Concentration and Downregulated the Superoxide Dismutase in HeLa and HCT 116 Cells

**Figure 12:**
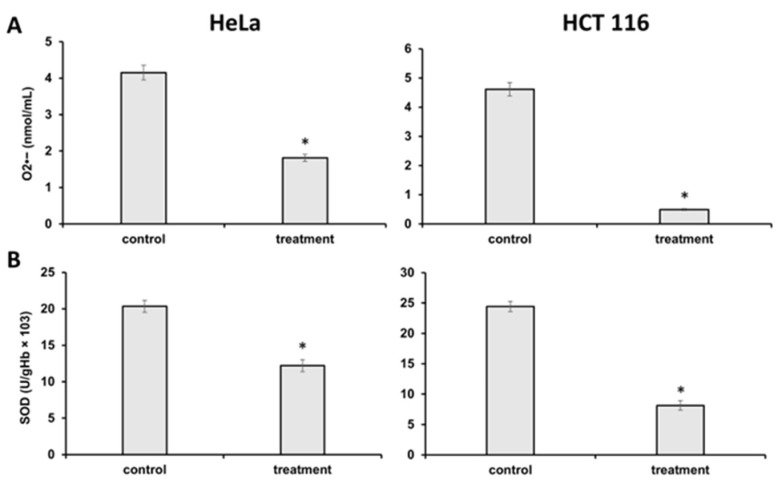
(**A**) Concentration of superoxide anion radical after treatment with IC_50_ values and a 48 h incubation in HeLa and HCT 116 cells. (**B**) Activity of superoxide dismutase after treatment with IC_50_ values and a 48 h incubation in HeLa and HCT 116 cells. Statistical significance compared to the control (non-treated) group—* *p* < 0.05.

In order to examine and confirm the in silico binding of secondary metabolites from *J. communis* EO to NDUFS1, NDUFS2, NDUFS6, and SOD, we performed in vitro experiments and measured the concentration of the superoxide anion radical (O_2_^−●^) and the activity of SOD. As shown in Figure 12A, in both the HeLa and HCT 116 cells, there was a statistically significant decrease in O_2_^−●^ concentration after the IC_50_ treatment (*p* < 0.05), as well as a decrease in the activity of the antioxidative enzyme SOD, *p* < 0.05 (Figure 12B).

## 3. Discussion

Cancer still poses a significant global health problem. An increasing number of multidrug-resistant cancers have been reported in recent years [1]. In that sense, finding novel therapeutic approaches is an interesting topic of research. Since the *Juniperus* genus essential oils had already been proven to have cytotoxic and antiproliferative effects [9,10,11], we wanted to specifically test the antitumor effect of *J. communis* berry EO on HeLa and HCT 116 cells. We showed that the *J. communis* berry EO showed selective short-term and long-term antitumor effects on the HeLa and HCT 116 cell lines. To the best of our knowledge, our findings represent the first confirmation of the anticancer effects of *J. communis* berry EO on HeLa and HCT 116 cells, including the triggering of the intrinsic pathway of apoptosis, alteration in the cell cycle, inhibition of cell-survival pathways, and changes in the oxidative status in cancer cells.

Numerous studies have demonstrated that EOs derived from *J. communis* variants induced cytotoxic, genotoxic, and antiproliferative effects in vitro [11,12]. Specifically, *J. communis* EO exhibited cytotoxic effects against the breast cancer MCF-7 cell line [9], neuroblastoma SH-SY5Y cells [10], and lung A549 cells [12]. Additionally, Mansour RB et al. [11] revealed the cytotoxic effects of *J. phoenicea* berry EO on breast MCF-7 and HT-29 colon cancer cells. Consistent with these findings, we demonstrated that the *J. communis* berry EO treatment is selective for the transformed cell lines HeLa and HCT 116, exhibiting both short-term and long-term cytotoxicity. This is corroborated by the fact that the predominant component in the EOs studied, including ours, is α-pinene, which is well-known for its cytotoxic properties [9,10,11,12].

One of the desirable outcomes of antitumor therapy is targeting the induction of programmed cell death. Apoptosis is a highly regulated physiological process that is necessary for the normal functioning of cells on a molecular level. It is characterized by cell membrane “blebbing”, chromatin condensation, nuclear fragmentation, and cell shrinkage without consequent inflammation and damage to neighboring cells, unlike necrosis, which is a type of cell death that causes the uncontrolled release of inflammatory cellular contents that results in inflammation and tissue damage. Apoptosis can be triggered via one of two primary pathways—the intrinsic and the extrinsic pathway. In the intrinsic pathway, apoptosis is initiated internally within the cell in response to cellular stress or damage, involving the alteration in mitochondrial membrane potential, the release of cytochrome C, and the activation of caspases. In contrast, the extrinsic pathway is activated by external signals, typically involving the binding of death ligands [13,14]. Many cancerous diseases are closely associated with the abnormal regulation of apoptotic pathways. One of the defining features of multidrug-resistant cancers is the ability of tumor cells to avoid apoptosis. To that extent, compounds that target apoptotic pathways and are highly selective to cancer cells raise great research interest [13,15]. In recent years, compounds of natural origin have been the focus of scientific research. To further test that hypothesis and explore the molecular mechanism of cell death on HeLa and HCT 116 cells, we performed a flow cytometric analysis of apoptosis and found that *J. communis* EO indeed triggered apoptotic pathways on both tested cell lines. In addition, we detected a substantial increase in cleaved caspase-3, a point-of-no-return executor caspase on both cell lines, which was unequivocal confirmation of the induction of apoptosis [16]. To determine the pathway of apoptotic death induced by *J. communis* EO, we performed an in-depth analysis of the proteins and events involved in the apoptosis. First, the pro-apoptotic Bax/antiapoptotic Bcl-2 protein ratio was higher in both the HeLa and HCT 116 cell lines under treatment, implying the intrinsic pathway of apoptosis. The intrinsic, known as mitochondrial, pathway of apoptosis is characterized by the alteration of mitochondrial membrane potential—a marker of mitochondrial dysfunction. JC-10, as a mitochondrial-membrane-potential probe, accumulates as red fluorescent aggregate within the mitochondria of a healthy cell. However, in cells undergoing apoptosis, JC-10 migrates from the mitochondria into the cytoplasm, where it converts into a monomeric form and gives a green fluorescence [17]. In the treated cells, we detected a statistically significant increase in green fluorescence—JC-10 was dispersed throughout the cell’s cytosol (Figure 3). In addition, one of the important steps of this pathway is cytochrome C release from mitochondria to the cytosol after mitochondria membrane damage, which plays a crucial role in the activation of caspase-3 [16]. In our experiments, cytochrome C shifted from mitochondria to cytosol in the treated samples (Figure 4). The evidence from the conducted experiments strongly suggests that the *J. communis* EO induced the intrinsic pathway of apoptosis. Additionally, we detected the inhibition of cell-survival signal pathways in HCT 116 cells, which directly induces apoptotic cell death, and to the best of our knowledge, this is the first piece of information about *J. communis* EO interfering with cell-survival pathways. These findings can be supported by the fact that α-pinene and limonene, highly present compounds in the tested EO, have known apoptotic effects on various cell lines [18,19,20], and specifically, Huang X et al. [21] showed the induction of the intrinsic pathway of α-pinene on the HeLa cell line, which closely correlates with our findings. Moreover, EO containing high levels of limonene induced apoptosis and reduced ΔΨm in HeLa cells [20]. These findings can suggest the synergistic effect of more than one active metabolite from *J. communis* EO.

The induction of apoptosis in tumor cells is often associated with DNA damage and cell-cycle imbalance [22]. Our results indicated that *J. communis* EO induced cell-cycle arrest in the S phase in HeLa cells, subsequently leading to apoptosis. This suggests that the treatment may cause significant DNA damage or the depletion of nucleotides, which typically forces cells into an S-phase arrest [16]. Interestingly, the G0/G1-phase arrest and consequent apoptosis were observed on the HeLa cell line treated with a beta-pinene-based derivative, a secondary metabolite also present in *J. communis* EO [23]. In contrast, a slight change in the cell-cycle distribution was observed in HCT 116 cells, indicating the possible arrest in the G2/M phase, consistent with the findings of Si C et al. [20], which highlighted limonene as the predominant secondary metabolite in the EO treatment, and Xu Q et al. [24], who studied alpha-pinene effects, whereby both reported G2/M-phase arrest in HeLa and hepatocellular carcinoma cells, respectively.

**Figure 13:**
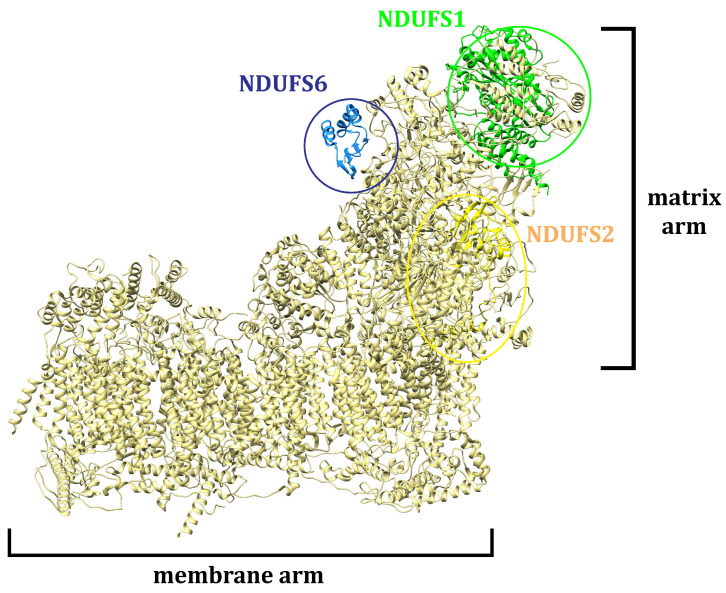
Structural characteristics of the CI enzyme.

The potential for *J. communis* EO components to interfere with the action of NADH ubiquinone oxidoreductase (complex I—CI) was assessed by molecular docking. As an L-shaped multisubunit assemblage, CI represents the largest enzyme of the respiratory chain, catalyzing electron transfer processes and proton translocation (Figure 13) [25].

Structurally, CI comprises matrix and membrane moieties (arms), further divided into N-, Q-, and P-modules [26]. The N- and Q-modules in the matrix arm are engaged in electron transfer, whereas the P-module maintains the proton translocation through the membrane arm [26]. From a molecular perspective, CI serves as the main entry point for the electrons toward the electron transport chain (ETC), and it is crucial in energy consumption. Cancer mitochondria metabolism can differ from that in normal cells due to increased energy demands making them more sensitive to changes in the ETC perturbation [27]. In this respect, the search for innovative small-molecule CI inhibitors for cancer treatment is still ongoing [28,29]. In this study, according to previous chemical characterization [30], the constituents present in the amount ≥5% were considered as major ones, whereas all others were observed as minor. The estimation of the binding potential was based on the calculated binding-affinity values and interaction pattern analysis, highlighting the three compounds with the highest binding affinity from both minor and major groups (Figure 8, Figure 9, Figure 10 and Figure 11). Generally, the obtained in silico results indicated the potential of EO constituents to favorably interact with CI. Based on the binding-affinity values, the investigated compounds expressed the highest binding potential to the NDUFS1 subunit of CI; however, the results regarding NDUFS2 and NDUFS6 should not be neglected. The literature data exposed the ability of *E. uniflora* EO to inhibit the electron transfer system and promote a collapse of the ΔΨm [31]. Furthermore, the findings reported by Dugue et al. suggested the inhibitory potential of the EOs of some American native plants against mitochondrial enzymes, including CI [32]. Here, among the top-ranked EO components against the investigated mitochondrial targets were also trans-caryophyllene, limonene, sabinene, and α-pinene [32], which is in agreement with the results of this study.

As a central subunit of the N-module, NDUFS1 is responsible for the oxidation of NADH to NAD^+^, whereas NDUFS2, as a part of the hydrogenase module (Q-module), is involved in the transfer of electrons to ubiquinone [33]. On the other hand, NDUFS6 is an auxiliary subunit found to be essential for CI biogenesis and stability [34,35]. It is indicated that the alterations of NDUFS6 could prevent the assembly of CI or destabilize the peripheral arm, leading to CI deficiency [35]. NADH oxidation by CI is important in generating the ΔΨm that supports ATP synthesis [36], and its inhibition can lead to mitochondrial depolarization [37]. In our experiments, the potential inhibition of CI led to the dissipation of the ΔΨm and the induction of the intrinsic pathway of apoptosis proven by the migration of cytochrome C from mitochondria to cytosol (Figure 4). These findings are in concordance with the findings of Lim SC et al., who demonstrated the inhibition of CI with isoflavan analogs and the subsequent loss of the ΔΨm [38]. Moreover, ETC and specifically CI is the main site of electron leakage and the production of O_2_^●−^ [39]. At the normal amount, O_2_^−●^ can serve as a signaling molecule in various cancer cell pathways [40]. Our molecular docking analysis revealed the potential inhibition of CI, and in vitro results confirmed the lower concentration of O_2_^−●^ in the treated cells. Additionally, a docking analysis was performed on SOD, an antioxidant enzyme that modulates the cell redox status. In physiological conditions, SOD functions as a defender from harmful ROS by converting O_2_^●−^ to H_2_O_2_ and O_2_. Similarly, SOD overexpression in tumors protects cancer cells [41,42]. In silico results revealed the favorable binding modes of *J. communis* EO components to SOD in comparison to the standard inhibitor LSC-1 (−5.7 kcal/mol), thus indicating the potential inhibitory activity on SOD. The obtained data were supported by the in vitro results, which showed a significant decrease in SOD activity in both cell lines after the treatment.

## 4. Materials and Methods

### 4.1. Cell Lines

MRC-5, the non-transformed human fetal lung fibroblast cell line; HeLa, the transformed human cell line from cervical adenocarcinoma; and HCT 116, transformed human colorectal carcinoma cells acquired from American Type Culture Collection (ATCC, Manassas, VA, USA) were grown in 25 cm^2^ flasks in Dulbecco’s Modified Eagle Medium (DMEM) (D5671, Sigma-Aldrich, St. Louis, MO, USA) with 10% heat-inactivated fetal bovine serum (FBS) (ECS5000L, EuroClone, Pero, Italy), non-essential amino acids (Sigma-Aldrich, M7145), L-glutamine (ECB3000D, EuroClone), and penicillin and streptomycin (P4333, Sigma-Aldrich), ensuring a suitable environment for cell growth. The cells were cultured under standard conditions in a sterile environment at 37 °C and 5% CO_2_ in a humidified incubator. Upon reaching 70% confluency cells were subcultured with a solution of 0.05% trypsin (6502, Sigma-Aldrich) and 0.053 mM EDTA.

### 4.2. Compound Solutions

The essential oil used in this study was acquired from the Hanus s.r.o. (Nitra, Slovakia). Based on the data provided by the manufacturer, the tested EO originated from Slovakia and was obtained by steam distillation of dried unfermented berries. Chemical characterization was performed by Cmikova N et al. in a previously published paper [30].

*J. communis* berry EO was dissolved in dimethyl sulfoxide (DMSO), making a stock solution (50 mg/mL). Working concentrations were made with the stock solution dissolved in DMEM—0.1, 0.3, 1, 3, 10, 30, and 100 µg/mL, using complete DMEM as the control in experiments. The concentration of DMSO did not surpass 0.2% in the highest working concentration.

### 4.3. Cytotoxicity of Juniperus communis Essential Oil

Cells were plated into 96-well microtiter plates. Each well was seeded with 3 × 10^3^ cells. Following the overnight adhesion, the cells were treated with prepared concentrations of *J. communis* EO (0.1–100 µg/mL) and incubated for 24, 48, and 72 h. The cytotoxic effect of *J. communis* EO was determined with a 3-(4,5-dimethylthiazol-2-yl)-2,5-diphenyltetrazolium bromide (MTT) assay according to Mosmann [43]. After dissolving the formazan crystals in DMSO, the absorbance was measured with the multiplate reader (BioTek Epoch Microplate Spectrophotometer, Agilent) at a wavelength of 540 nm. The experiment was repeated in triplicates and the results of the absorbance values (A) were presented as mean values. The percentage of cytotoxicity was calculated using the following standard equation:

((A_control_ − A_treatment_)/A_control_) × 100 (1)

Cytotoxicity was presented as the mean value from three separate experiments ± standard deviation. The IC_50_ value (50% inhibitory concentration) was calculated with GraphPad Prism 8.0 software. The selectivity index (SI) was calculated as MRC-5 mean IC_50_ value/HeLa or HCT 116 mean IC_50_ value, and the percentage growth (GI_50_, TGI, and LD_50_) was calculated following the equations given by the National Cancer Institute NCI-60 Screening Methodology [44].

### 4.4. Clonogenic Assay

To assess the long-term effect of *J. communis* EO on cytotoxicity and the ability to form colonies, the HeLa and HCT 116 cell line was plated into 6-well plates at an initial density of 100 cells per well. Following, an overnight incubation, the cells were treated with *J. communis* EO at concentrations of 0.3, 1, 3, 10, and 30 µg/mL. After treatment, the cells were incubated in a CO_2_ incubator at 37 °C for twelve days, with fresh complete DMEM added on every fourth day.

Following the incubation period, colonies were washed with PBS, fixed in ice-cold methanol for 15 min, and stained with a fresh filtered 10% Giemsa solution for 30 min at room temperature (RT). The colonies were manually counted using an inverted fluorescent microscope (FLUO500, Colo Lab, Ljubljana, Slovenia). Groups containing more than 50 cells were considered as individual colonies. The results are presented as the SF representing the proportion of cells that survived the treatment. The SF was calculated as the number of colonies formed after treatment divided by the number of cells seeded multiplied by the plating efficacy (PE), where the PE is the ratio of the number of colonies formed in control wells to the number of cells initially seeded [45].

### 4.5. Flow Cytometry Analysis of Apoptosis, Autophagy, and Protein Expression

The HeLa and HCT 116 cell lines were plated in 24-well microtiter plates at a density of 1 × 10^5^ cells per well and incubated for 24 h. Subsequently, the cells were treated with calculated IC_50_ values of *J. communis* berry EO and incubated for 48 h for every analysis. Early apoptosis, late apoptosis, and necrosis were quantified using the Annexin V-FITC/7-AAD kit (IM364, Beckman Coulter, Brea, CA, USA) following the manufacturer’s instructions. In brief, after treatment, the cells were collected, washed in PBS, and suspended in an ice-cold binding buffer, constantly keeping the cells on ice. The cells were then stained with 10 µL of Annexin V-FITC and 20 µL of 7-AAD and incubated for 15 min at +4 °C in the dark. Before measuring, 400 µL of binding buffer was added to each test tube.

For protein expression, the HeLa and HCT116 cell lines were seeded in 24-well plates at a density of 10^5^ cells per well and incubated overnight in a CO_2_ incubator. The cells were then treated with the corresponding IC_50_ values of *J. communis* EO and left to incubate for 48 h. After incubation, the cells were collected and fixed in 4% paraformaldehyde for 30 min, permeabilized with 0.2% Tween 20 in PBS, incubated with specific primary antibodies (1:100)—mouse antihuman active caspase-3 (9661, Cell Signaling, Danvers, MA, USA), 1:100 mouse antihuman Bax (2D2, sc-20067, Santa Cruz Biotech. Inc., Dallas, TX, USA), mouse antihuman-Bcl-2 (DC21, sc-783, Santa Cruz Biotech. Inc.), Phospho-Akt (Ser473, 9271, Cell Signaling), and Phospho-Erk (D13.14.4E, 4370, Cell Signaling)—and then incubated with the secondary antibody mouse antihuman FITC (1:200).

For each sample, 10.000 cells were analyzed on Cytomics FC500 (Beckman Coulter), and the results were calculated using FlowJo™ v10 software. The results are represented as the mean values ± SD from three separate experiments.

### 4.6. Cell-Cycle Analysis

The HeLa and HCT 116 cell-cycle distribution was assessed through flow cytometry. Briefly, the cells were seeded in 24-well microtiter plates (a density of 1 × 10^5^ cells per well) and then treated and incubated with the IC_50_ values for 48 h. Then, the cells were harvested, washed in PBS, fixed with ice-cold ethanol, and incubated at +4 °C for 24 h. The fixed cells were re-suspended and incubated with 1 mL of RNaseA (500 µg/mL in PBS) for 30 min at RT. After incubation, 5 µL of propidium iodide (10 mg/mL in PBS) was added and incubated for 15 min in the dark. The DNA content was measured on Cytomics FC500 (Beckman Coulter).

### 4.7. JC-10 and Cytochrome C Localization and Quantification

The HeLa and HCT116 cells were incubated overnight at a density of approximately 10^4^ cells per well in 24-well cell culture plates. The *J. communis* EO IC_50_ value was added to the cells and incubated for 48 h. For the JC-10 experiment, the cell culture medium was discarded after incubation and the cells were treated with 2.5 µM JC-10 dye in warm PBS at 5% CO_2_ and 37 °C for 20 min and then rinsed once with warm 1xPBS. For the cytochrome C experiment, the cells were fixed for 30 min at RT in paraformaldehyde (4%), permeabilized with Tween 20 (0.2%) in PBS, and blocked in Tween 20 (0.1%) in PBS for 30 min and then incubated for 60 min at RT with a monoclonal antimouse human cytochrome c antibody (G7421, 1:100, Promega, Madison, WI, USA). After a 30 min incubation with goat antimouse FITC (1:200) diluted in a blocking solution, the cells were washed with PBS. The cells were examined using an inverted fluorescent microscope (FLUO500, Colo Lab). ImageJ 1.8.0 software was used to examine the images after they were taken by the 2020 S-EYE Setup Microscope camera using the S-EYE_Setup-1.6.0.11 software. For the quantification analysis, the ratio of fluorescence emissions at 525 and 590 nm was employed.

### 4.8. Molecular Docking Screening

A thorough in silico screening of *J. communis* EO components with mitochondrial complex I—NADH ubiquinone oxidoreductase (PDB ID: 5xtd) and superoxide dismutase (PDB ID: 4mcm) was conducted utilizing AutoDock Vina software 1.2 [46,47,48]. Before the docking analysis, the protein structures were appropriately prepared using the Chimera 1.17 program [49]. Initially, the geometries of the ligand molecules were optimized using the Density Functional Theory (DFT) B3LYP functional in conjunction with the 6–311 + G(d, p) basis set [50,51,52]. Also, the ligands were subjected to conformational analysis using VeraChem’s Vconf 2.0 (VeraChem LLC, Germantown, MA, USA). Here, the ligand structures within a 5.0 kcal/mol threshold were used for molecular docking. In addition, active-site pockets were predicted using the CastP online tool (http://sts.bioe.uic.edu/castp/ (accessed on 23 May 2024) [53]). The grid parameters for 4mcm were set such that the whole protein was set within the cuboid grid box (center x, y, z = 24.5, 122.7, 13.7; size x, y, z = 40.0, 31.7, 41.4). In the case of 5xtd, a docking analysis was performed on the NADH ubiquinone oxidoreductase core subunits S1, S2, and S6 (NDUFS1, NDUFS2, and NDUFS6, respectively), setting the grid parameters as follows: for NDUFS1 center x, y, z = 211.5, 142.3, 364.3, size x, y, z = 24.0, 24.0, 24.0; for NDUFS2 center x, y, z = 215.0, 160.0, 297.0, size x, y, z = 28.0, 37.0, 41.0; for NDUFS6 center x, y, z = 214.0, 218.0, 350.0, size x, y, z = 36.0, 33.0, 31.0. The BIOVIA Discovery Studio Visualizer was employed for the analysis of the ligand–macromolecule interaction patterns, as well as for the preparation of 2 d and 3 d graphical interpretations [54].

### 4.9. Determination of Superoxide Anion Radical Concentration and Superoxide Dismutase Activity

The concentration of O_2_^−●^ and the activity of SOD were measured by the Agilent BioTek Epoch Microplate Spectrophotometer (Agilent Technologies, Santa Clara, CA, USA). O_2_^−●^ was quantified after the reaction of nitro blue tetrazolium in a TRIS (tris(hydroxymethyl)aminomethane) buffer (an assay mixture) with the cell supernatant at 530 nm. The cell lysate was prepared in water with repetitive freezing and thawing of the samples. In the prepared lysate, the activity of superoxide dismutase SOD was determined using the epinephrine method. In a mixture of lysate and carbonate buffer, epinephrine was added, and the detection was performed at a wavelength of 470 nm.

### 4.10. Statistical Analysis

All the experiments were repeated three times in triplicates, and the results are presented as the mean values ± standard deviations. Considering the number of groups for comparison, a one-way ANOVA or paired sample *t*-test was employed. A *p* value lower than 0.05 was considered significant.

GraphPad Prism^®^ 8.0 was used for all the statistical analyses. Microsoft Excel^®^ (version 2013) as well as GraphPad Prism^®^ 8.0 was used for the graphical presentation of the data.

## 5. Conclusions

In this study, we demonstrated the anticancer molecular mechanisms of *J. communis* berry essential oil in cervical and colorectal cancer cells. Our results show the potential of the essential oil as an anticancer agent through both in vitro and in silico analyses. Elevated levels of the Bax/Bcl-2 ratio and cytochrome C migration to cytosol observed in vitro and the inhibition of NDUFS1, NDUFS2, and NDUFS6 observed in computational studies indicate the disruption of the mitochondrial membrane potential and initiation of the intrinsic pathway of apoptosis. In addition, our findings reveal the potential of *J. communis* berry essential oil to interfere with the cell cycle and cell-survival pathways.

## Figures and Tables

**Table 1:** The concentration of *J. communis* EO (µg/mL) that induces inhibition of biological activity in 50% of cells, selectivity index, 50% growth inhibition, total growth inhibition, and 50% lethality in the MRC-5, HeLa, and HCT 116 cell lines.

Cell Line	Incubation Time (h)	IC_50_ ± SD	SI	GI_50_ ± SD	TGI ± SD	LD_50_ ± SD
MRC-5	24	88.02 ± 28.00	NA	36.98 ± 11.32	72.23 ± 22.58	>100
	48	34.86 ± 5.32	NA	31.33 ± 4.61	59.79 ± 9.69	>100
	72	27.88 ± 3.19	NA	21.75 ± 7.21	48.26 ± 8.59	99.51 ± 18.91
HeLa	24	15.84 ± 4.20 *	5.56	12.18 ± 0.86	21.55 ± 0.77 *	>100
	48	10.14 ± 2.89 *	3.44	8.04 ± 3.91 *	17.35 ± 7.55 *	>100
	72	10.29 ± 2.02 *	2.71	9.02 ± 2.93	20.43 ± 2.04 *	31.12 ± 0.04 *
HCT 116	24	40.51 ± 34.59	2.17	34.63 ± 16.43	61.51 ± 16.40	74.02 ± 38.82
	48	29.19 ± 6.12 ^	1.19	30.71 ± 12.49 ^	52.54 ± 21.89	74.88 ± 27.03
	72	25.25 ± 9.96	1.10	26.80 ± 9.29 ^	50.62 ± 18.64 ^	73.91 ± 23.06 ^

Statistical significance: MRC-5 compared to HeLa—* *p* < 0.05; HeLa compared to HCT 116—^ *p* < 0.05. NA—non applicable.

## Data Availability

Unprocessed data will be available upon request to the corresponding author.

## Supplementary Materials

The following supporting information can be downloaded at https://www.mdpi.com/article/10.3390/plants13172351/s1: Figure S1: Dose–response curves determined by the MTT assay following the treatment of cells with *Juniperus communis* essential oil; Figure S2: Dose–response curves determined by the MTT assay following the treatment of cells with doxorubicin.
