# Supplementary material for: Insights into Molecular Mechanisms of Anticancer Activity of Juniperus communis Essential Oil in HeLa and HCT 116 Cells
Source: Plants (Basel). 2024 Aug 23;13(17):2351. doi: 10.3390/plants13172351 (PMC11397105; doi:10.3390/plants13172351)
Supplement: Supplementary file 1 [file plants-13-02351-s001.zip › plants-3136046-supplementary.pdf]

plants

Supplementary Material

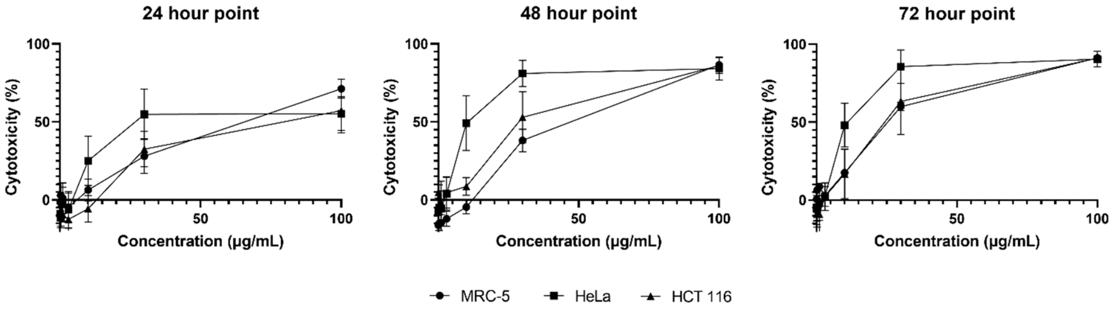

Figure S1. Dose-response curves determined by the MTT assay 24, 48, and 72 hours following the treatment of cells with *J. communis* EO. Results were presented as mean ± SD cytotoxicity percentages from three separate experiments.

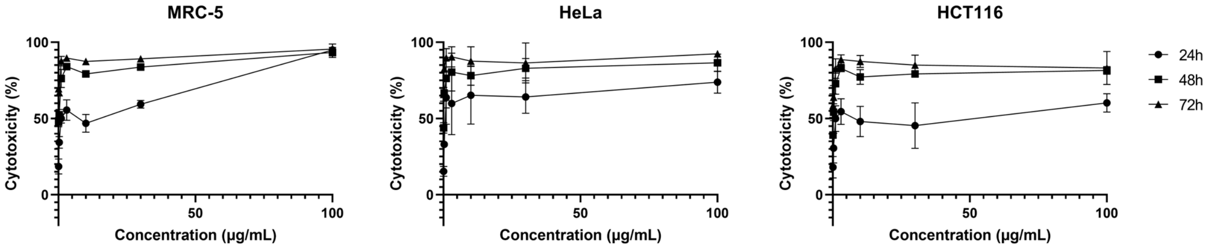

Figure S2. Dose-response curves determined by the MTT assay 24, 48, and 72 hours following the treatment of cells with doxorubicin. Results were presented as mean ± SD cytotoxicity percentages from three separate experiments.
